# A Rare Case of Recurrent Hematometra of Unknown Etiology

**DOI:** 10.7759/cureus.29217

**Published:** 2022-09-15

**Authors:** Sonal Gupta, Vaishali Ambatkar, Kanan Kotdawala

**Affiliations:** 1 Obstetrics and Gynaecology, Acharya Vinoba Bhave Rural Hospital, Jawaharlal Nehru Medical College, Datta Meghe Institute of Medical Sciences, Wardha, IND

**Keywords:** bilateral salpingo-oophorectomy, total abdominal hysterectomy, usg guided hematometra drainage, gnrh analogue, scar adhesion, caesarean section, hematometra

## Abstract

An abnormal blood collection in the uterus is referred to as hematometra. Obstruction of the genitourinary outflow system caused by earlier surgeries or congenital defects is most frequently related to this rare disorder. The symptoms of hematometra include acute pelvic pain and a history of absent menarche. Here is a case of a 42-year-old female who presented with complaints of severe lower abdominal pain, and pain during urination that was accompanied by vulval itching in June 2021. She had undergone two Caesarean sections and a myomectomy in the past. She was given three monthly injections of gonadotropin-releasing hormone (GnRH) analogue after receiving USG-guided drainage because of a diagnosis of hematometra in January 2021. However, in June 2021, she experienced a recurrence of the same symptoms, necessitating a total abdominal hysterectomy and bilateral salpingo-oophorectomy, which completely resolved the patient's complaints. For a deeper understanding of this issue, further case reporting is necessary.

## Introduction

Hematometra, by definition, is the pathologic accumulation of blood in the uterus [1]. It can also be defined as a collection of menstrual fluid inside the uterus. It can occur in premenarchal, reproductive age, as well as post-menopausal females. It may be due to certain congenital anomalies such as transverse septum of the uterus, stenotic cervix or atresia of the cervix; or blockage of the genitourinary outflow tract, due to previous surgeries.

With fewer than 100 occurrences reported in the literature, cervical agenesis is an infrequent Mullerian defect. The vagina and a functioning uterus are infrequently linked to cervical agenesis. Hematometra will happen if there is an association with the functioning uterus [2]. Here is a presentation of a case of hematometra, of unknown etiology, in a patient with previous history of two caesarean sections. Out of several probable causes of hematometra, one may be scar adhesion (uterine cavity scar formation is referred to as Asherman’s syndrome). This is a report of a rare and important case due to the presence of recurrent hematometra, in which recurrence occurred even after ultrasound-guided drainage of hematometra.

Symptoms in association with hematometra consist of amenorrhea, dysmenorrhea in premenopausal women, pelvic pressure or pelvic pain, tenesmus, and retention of urine [3]. Lower abdomen may swell and tenderness may also be present. There may be evidence of infection, which may present as fever. According to some sources, infertility can manifest in women in addition to pregnancy issues such as repeated miscarriages, premature delivery, and malpresentation. Hence, early diagnosis is crucial [4]. Pyometra, in simple words, is the collection of pus in the uterus, which may also be due to cervical stenosis, like hematometra. A purulent collection inside the uterus is referred to as pyometra. One unusual consequence of pyometra is spontaneous perforation [5]. Differential diagnosis of hematometra are pyometra, hydrometra and hematometrocolpos. Hematometrocolpos is a condition in which the uterus and vagina both are filled with blood [6]. An intravenous pyelogram (IVP) may provide assistance in the diagnosis of a particular congenital anomaly that may serve as the underlying cause of the hematometra. The ultrasound technique uses high frequency sound waves for creating the images of the interior of the body. It is suitable for confirming hematometra. Hematometra has also been reported as an uncommon complication of endometrial ablation [7]. There are many more causes of hematometra, and new correlations are being formed in research.

## Case presentation

A 42-year-old female, para-2, live-2, came to our hospital on June 18, 2021, with complaints of severe pain in the lower abdomen for about six years (on and off), and pain during micturition associated with vulval itching. She had a history of two caesarean sections in 2003 and 2006; one was low segment C-section and another was a vertical C-section. Unilateral tubal ligation was done in 2007 due to dense adhesions on one side. She also underwent a laparoscopic myomectomy in 2018. The patient had her first visit in January 2021 with similar complaints. On ultrasonography, it was found that she had constricted endocervical canal with hematometra of approximately 20 cc in size. At that time, on January 29, ultrasound-guided drainage of hematometra was done. After that, the patient was given injectable gonadotropin-releasing hormone (GnRH) analogue (three leuprolide injections) for three continuous months (one month apart each), i.e. in January, February, and March. After that, her pain subsided and she had no complaints.

In third week of June, 2021, the patient developed pain in groin region, which was insidious in onset, radiating along the inner thigh, severe in intensity. Ultrasonography and contrast-enhanced computed tomography (CECT) showed hematometra of about 30 cc. As the patient was nearing menopause, she needed a permanent cure and, hence, the decision of hysterectomy was taken. General Surgeon was also consulted as many postoperative adhesions were suspected. After necessary investigations, a negative report for coronavirus disease 2019 (COVID-19), and informed consent from the patient, surgery (total abdominal hysterectomy and bilateral salpingo-oophorectomy) was planned for June 21, 2021.

**Figure 1:**
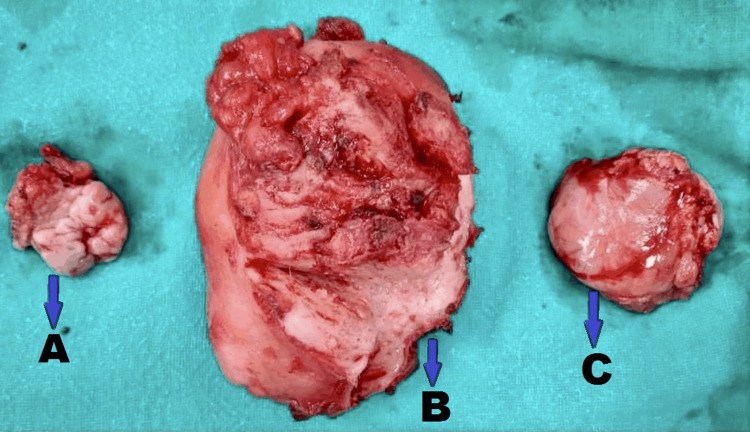
Bulky uterine specimen with hematometra and constricted endocervical canal with bilateral salpingo-oophorectomy: (A) right ovary with fallopian tube adhered; (B) uterus with cervix; (C) left ovary with fallopian tube adhered

The patient was induced with spinal and epidural anaesthesia and a Foley catheter was placed. The abdomen was opened in layers, the rectus sheath was cut, and the muscles were separated. Uterus along with bilateral fallopian tubes and bilateral ovaries were identified. There were many adhesions during the operative period. Kelly clamps were placed on bilateral round ligament, clamped, cut, and ligated. A window was created between the infundibular pelvic and ovarian ligament. Bilateral infundibulo-pelvic ligament was clamped, cut, and ligated. Retractors were placed into the incision and bowel was packed away with help of moist laparotomy sponges. Then the uterus was held with straight clamps and the anterior leaf or broad ligament was incised toward the bladder reflection to the midline from both sides with Metzenbaum scissors, and the ureterovesical fold was separated. The bladder was gently dissected off the lower uterine segment with a combination of sharp and blunt dissection. The uterine arteries were skeletonised bilaterally clamped, cut, and ligated. Bilateral uterosacral ligaments with Mackenrodt's ligament were clamped, cut, and ligated. The uterus, bilateral fallopian tubes, and ovaries were removed. On cutting the uterus, there was blood collection (hematometra) noted in the uterine cavity. The uterus, ovaries and fallopian tubes were sent for histopathological investigations. The postoperative period was uneventful. The patient was relieved of all her complaints and was discharged on postoperative day eight. The bulky uterine specimen with hematometra and constricted endocervical canal with bilateral salpingo-oophorectomy is shown in Figure 1.

## Discussion

It has been estimated that postoperative hematometra occurs in 1-2% of all females who undergo endometrial resection or endometrial ablation [8]. In general, this complication should be taken into consideration if a female is presenting from 2-15 months postoperatively with cyclic, midline, cramp, and amenorrhea (likely). Diagnosis is made by ultrasonography, but examination might exhibit cervical stenosis also. Management options include the use of GnRH agonists, drainage of hematometra, and hysterectomy. Hematometra and intra-uterine adhesions are long-term complications of the caesarean section mode of delivery. Complications of hematometra may include infection because the retained tissue may act as foci of infection. Pelvic pain needs to be ruled out immediately, because it may be pointing towards the diagnosis of hematometra. Recurrence of hematometra, even after ultrasonography-guided drainage, as in this case, also seems to be a complication; a complication is defined as a condition that develops after a medical procedure, or a disease. Hematometra has been seen to be associated with anaemia in some case reports of dogs [9]. Human studies should be conducted to find out whether anaemia could be a complication in cases of hematometra because this could then be a rare cause of anaemia in females.

Management

Hematometra is a significant cause of pelvic pain in women. Management includes simple cervical dilation but, at times hysterectomy may be required. Another option for managing the condition is the use of GnRH analogues. However, if they fail, drainage of hematometra or hysterectomy may be needed. GnRH agonists are synthetic peptide molecules that interact with the GnRH receptors to elicit the biological response by stimulating the release of pituitary hormones, follicle-stimulating hormone (FSH), and luteinizing hormone (LH). Since the physiological release of GnRH is in pulses, whereas the GnRH agonists act continuously, soon after the initial episode of stimulation, they produce a hypogonadotropic-hypogonadic state. They do so by inhibiting the secretion of gonadotropins (by the down-regulation of the pituitary gland). After an initial episode of stimulation, and causing the down-regulation of the pituitary gland, GnRH agonists produce a hypogonadotropic-hypogonadic state [10]. Another mechanism by which they act is the inhibition of the mid-cycle FSH and LH surge and, hence, by preventing steroidogenesis in the corpus luteum. Spermatogenesis or ovulation comes to an end and testosterone or estradiol levels fall to the castration levels. Recovery is expected within a time frame of two months of stopping treatment. Currently, goserelin and leuprolide acetate are one of the most commonly used GnRH analogues [11]. They are used primarily as preoperative therapy in cases of fibroid. It has been demonstrated how their use can improve both preoperative and postoperative results, and shorten the duration of hospital stay. These outcomes are achieved by the action of GnRH agonists in decreasing menstrual bleeding (and hence uterine volume) by approximately 50% [12].

Hematometra can be diagnosed by pelvic ultrasound. A female with pelvic pain and previous history of surgical procedures could present with hematometra because of complications of the surgical procedure, like adhesions. Hysterectomy (definitive treatment) and bilateral salpingo-oophorectomy can both be performed during a single procedure. This surgery removes the cervix, ovaries, uterus, and oviducts. After the hysterectomy, a female will no longer have menstruation or be able to conceive [13].

## Conclusions

Hematometra can lead to the formation of a mass; the uterus may undergo distortion or may lead to cervical bulging. If the patient presents to the emergency department with pelvic pain, hematometra can be considered based on obstetrical and gynaecological history. Early diagnosis and treatment can prevent severe complications of hematometra. However, this requires the patient to be vigilant and aware, which is difficult in rural populations. If the patient approaches the doctor even with mild symptoms, she could avoid possible complications. Hence, even the slightest symptoms should not be ignored. Also, for rural populations, awareness needs to be created by healthcare professionals. Patients should be aware of the fact that any postoperative pain or other symptoms after any minor or major surgery or illness should not be ignored, and they should seek immediate help.
